# Complete Genome Sequence of Glutamicibacter sp. Strain M10, Isolated from an Arctic Permafrost Sample

**DOI:** 10.1128/mra.01120-22

**Published:** 2023-01-23

**Authors:** Polina Guro, Ekaterina Karaevskaya, Irina Kuznetsova, Denis Karlov, Anna Sazanova, Vera Safronova

**Affiliations:** a All-Russia Research Institute for Agricultural Microbiology, St. Petersburg, Russian Federation; b Arctic and Antarctic Research Institute, St. Petersburg, Russia Federation; SIPBS, University of Strathclyde

## Abstract

Permafrost is an extremely cold ecosystem that is inhabited by microorganisms with unique biochemical properties for potential biotechnological applications. Here, we present the complete genome sequence of *Glutamicibacter* sp. strain M10, which was isolated from a permafrost sample that had been collected at a depth of 2 m in West Spitsbergen, Norway.

## ANNOUNCEMENT

Strain M10 was isolated from the top layer of the permafrost, which in the Holocene Climate Optimum was an active topsoil layer (1). A core of permafrost sediments that had been obtained by drilling a borehole on the southern shore of Isfjorden Bay (78.09856°N, 14.23299°E; 43.0 m above sea level) was used. A suspension was prepared from the permafrost core sample; 0.4 g of the sample was suspended in 5 mL sterile 0.9% NaCl solution. Plating was performed in duplicate in Reasoner's 2A (R2A) medium (Difco, USA), by adding 100 μl of suspension to each petri dish and incubating the dishes at 5°C. To obtain a pure culture, one colony was selected and incubated in R2A broth for 48 h at 28°C. Genomic DNA was isolated using the DNeasy blood and tissue kit (Qiagen, Germany) according to the manufacturer's recommendations. The SQK-LSK109 ligation sequencing kit (Oxford Nanopore Technologies [ONT], UK) with the EXP-NBD104 native barcoding expansion 1-12 kit (ONT) was used to prepare the library according to the manufacturer’s instructions, skipping the DNA-shearing step. Long-read whole-genome sequencing was performed using a MinION sequencer with an R.9.4.1 flow cell (ONT). The reads were base called and demultiplexed using the Guppy (v3.3.0) base caller (2). There were 156,999 raw sequence reads, with an average length of 3,805 bp. Quality control of the raw reads was provided by NanoStat (v1.6.0) (3). For finding and removal of adapters from the ONT reads, Porechop (v0.2.1) (https://github.com/rrwick/Porechop) was used; the ONT reads were then assembled using Flye (v2.9) with two steps of polishing (parameter: –i 2) (4). Default parameters were used for all tools except for the Flye assembler. Two circular contigs, i.e., one chromosome of 3,529,731 bp and one plasmid of 11,885 bp, were assembled, with a total size of 3.54 Mbp and an average coverage of 168×. The quality of the assembly was evaluated with QUAST (v5.0.2) (5). The GC content was 55.64%, and the *N*_50_ value was 3,529,731 bp. The circular genome was obtained by the assembler without a specific starting point for the assembled contigs and was checked by NUCmer (v4.0.0rc1) for overlaps between the start and end positions using self-alignment (6).

Annotation using the NCBI Prokaryotic Genome Annotation Pipeline (PGAP) (v6.3) (7) predicted 3,426 total genes, 3,065 coding sequences, and 87 RNA genes (19 rRNAs, 65 tRNAs, and 3 noncoding RNAs). To identify isolate and phylogenetic relatedness, rRNA genes were predicted using Barrnap (v0.9) (https://github.com/tseemann/barrnap). The 16S rRNA gene sequence of *Glutamicibacter* sp. strain M10 was closely related (99.15% identity) to that of the type strain Glutamicibacter halophytocola KLBMP 5180 (GenBank accession number CP012750.1). Average nucleotide identity values with respect to the closest type strain, G. halophytocola KLBMP 5180, were calculated using the JSpeciesWS service and showed 79.70% identity calculated based on BLAST+ and 84.61% identity calculated based on MUMmer (8, 9).

### Data availability.

All data are available at GenBank under BioProject accession number PRJNA881974. The BioSample accession number is SAMN30921635. The raw MinION data can be found under SRA accession number SRR22371349, and the nucleotide accession numbers are CP104918.1 and CP104919.1.
